# The Role of NLR, PLR, SII and CRP Pre- and Post-Treatment with Infliximab in Rheumatoid Arthritis

**DOI:** 10.3390/biomedicines14010255

**Published:** 2026-01-22

**Authors:** Diellor Rizaj, Avni Kryeziu, Artidon Kelmendi, Behar Raci, Shend Kryeziu, Visar Baftijari

**Affiliations:** 1 Rheumatology Clinic, University Clinical Center of Kosovo, 10000 Prishtina, Kosovo or diellor.rizaj@ubt-uni.net (D.R.);; 2 Faculty of Medical Sciences, UBT College Higher Education Institution, 10000 Prishtina, Kosovo; 3 Clinic of Oncology, University Clinical Center of Kosovo, 10000 Prishtina, Kosovo; 4 Faculty of Medicine, University of Prishtina, 10000 Prishtina, Kosovo; 5 Clinic of Internal Medicine, University Clinical Center of Kosovo, 10000 Prishtina, Kosovo

**Keywords:** NLR, PLR, SII, CRP, arthritis rheumatoid, infliximab

## Abstract

**Background**: Inflammatory activity in rheumatoid arthritis can be determined by normal blood count ratios such as Neutrophil Lymphocyte Ratio (NLR), Platelet Lymphocyte Ratio (PLR), Systemic Immune Inflammation Index (SII), and C-reactive Protein (CRP). **Objective**: The aim of this research is to determine how these markers change after therapy and whether their pre- and post-treatment differences follow patterns that allow for simple parametric analyses. **Methods**: A prospective cohort of 52 RA patients (30 females and 22 males) was examined. The patients’ blood samples were tested at baseline and at the end of their 6-month Infliximab treatment. Hematologic markers such as NLR, PLR, and SII were calculated from the complete blood count (CBC), and CRP levels were measured. The statistical methods of Shapiro–Wilk (SW), Kolmogorov–Smirnov (KS), and Anderson–Darling (AD) were used, and later, paired *t*-tests were used to generate statistics where necessary. **Results**: Post-treatment measurements were consistently lower for all four biomarkers. QQ-plots and formal tests revealed that the differences between findings were essentially normal, allowing for paired *t*-tests. The mean decreases were as follows: NLR −1.10 (95% CI −1.48 to −0.71), PLR −43.0 (−55.4 to −30.7), SII −299 (−388 to −211), and CRP −11.36 (−13.18 to −9.54), all *p* < 0.001. CRP showed the greatest drop, with significant decreases in PLR and SII and a moderate decline in NLR, indicating therapy-related attenuation of systemic inflammation. **Conclusions**: The study shows that six months of infliximab therapy results in a consistent post-treatment decrease in all four biomarkers: NLR, PLR, SII, and CRP. Because the pre-post differences were roughly normal, CRP revealed the greatest decrease, with significant decreases in PLR and SII and a moderate decrease in NLR, consistent with systemic inflammation reduction. When combined, the CBC-derived indices track with CRP and can serve as practical, low-cost markers for monitoring therapy response in RA, despite the single-arm design.

## 1. Introduction

Rheumatoid arthritis (RA) is a systemic autoimmune disease affecting approximately 1% of the global population [1], characterized by chronic inflammation of the synovium, which leads to joint destruction, disability, and reduced quality of life [2,3]. The socioeconomic burden is significant, not only due to direct medical costs but also because of lost productivity and lifelong disability [4]. The World Health Organization (WHO) has identified RA as one of the leading causes of disability-adjusted life years (DALYs) among musculoskeletal disorders [5].

At the cellular level, RA pathogenesis involves infiltration of neutrophils, monocytes, and lymphocytes into the synovial fluid, with pannus formation and cartilage destruction [2]. Tumor necrosis factor-alpha (TNF-α), interleukin-6 (IL-6), and interleukin-1 (IL-1) are central cytokines that drive endothelial activation, leukocyte recruitment, and fibroblast-like synoviocyte proliferation [6]. Infliximab, a chimeric monoclonal antibody against TNF-α, has therefore become a cornerstone in RA therapy, disrupting these inflammatory pathways and inducing apoptosis of activated T cells [7]. Infliximab was selected as the biological agent in this study due to its well-established role as a TNF-α inhibitor with proven long-term efficacy and safety in rheumatoid arthritis. Furthermore, it remains one of the most widely used and clinically validated biologic options following inadequate response to conventional DMARDs. Its intravenous administration allows direct clinical supervision and optimized treatment adherence, supporting its continued use as a pragmatic standard-of-care therapy in RA.

Traditional biomarkers such as erythrocyte sedimentation rate (ESR) and C-reactive protein (CRP) remain widely used to monitor RA activity. However, their reliability can be limited by inter-personal variability, comorbidities, or financial cost in resource-limited settings [8]. This has encouraged the use of hematological indices derived from complete blood counts (CBC), including neutrophil-to-lymphocyte ratio (NLR), platelet-to-lymphocyte ratio (PLR), and the systemic immune-inflammation index (SII) [9]. These indices are cost-effective, reproducible, and increasingly validated across multiple chronic conditions [10].

The Disease Activity Score in 28 joints (DAS28) is a validated and widely used composite index for assessing disease activity in rheumatoid arthritis, particularly when calculated using C-reactive protein (DAS28-CRP).

The rationale for these indices lies in their ability to capture the balance between innate and adaptive immunity. NLR reflects neutrophil-driven inflammation versus lymphocyte-mediated regulation; PLR incorporates thrombocytosis as a marker of inflammatory stress; and SII integrates neutrophils, lymphocytes, and platelets into a composite index, providing a broader picture of systemic inflammation [11]. Importantly, these indices have demonstrated predictive and prognostic value not only in RA but also in oncology [12], cardiovascular disease [13], and systemic lupus erythematosus [14].

However, little evidence is available on how these indices behave specifically under biological treatment with infliximab, particularly in low-resource contexts such as Kosovo. This study, therefore, aims to investigate the pre-treatment and post-treatment values of NLR, PLR, and SII in RA patients receiving infliximab and to assess their correlation with CRP as a validation biomarker. The findings are expected to provide cost-effective, clinically relevant insights for RA monitoring, especially in regions where advanced laboratory markers are not widely accessible.

## 2. Materials and Methods

This is a prospective observational study conducted at the University Clinical Center of Kosovo from March 2023 to February 2024. There were 52 patients with established RA, according to the American College of Rheumatology (ACR) and European Alliance of Associations for Rheumatology (EULAR) 2010 criteria. The average age of the participants was 52.3 ± 12.7 years, with 30 females (57.7%) and 22 males (42.3%). For additional demographic details, see Table 1. The inclusion criteria were as follows: at least 18 years old, active RA despite prior use of disease-modifying anti-rheumatic drugs (DMARDs), and introduction to infliximab.

Any individuals with active infections, hematologic malignancies, other autoimmune diseases, and exposure to corticosteroids of more than 7.5 mg/day were excluded. Other exclusion criteria were uncontrolled hypertension, poorly controlled diabetes mellitus, and exposure to tuberculosis without prophylaxis. The patients were followed up monthly to monitor any ill effects and infusion reactions as well as clinical signs and symptoms of infection or disease flare. The patient compliance was confirmed by the electronic infusion log and nursing records.

In addition to blood samples, physical examinations were performed at each visit, and joint counts (tender and swollen) were noted but were not formally included in the current dataset.

**Figure 1:**
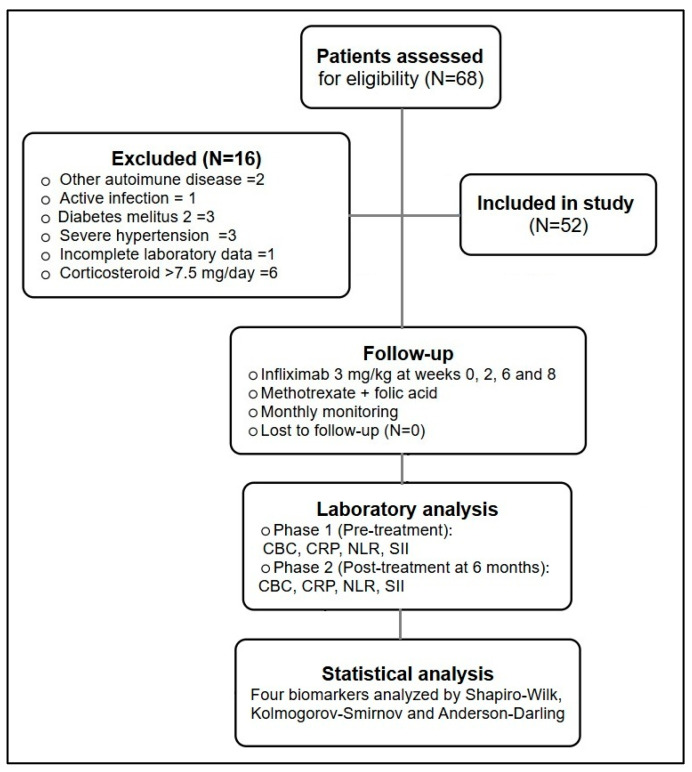
Diagram of patient selection and analysis process.

The flow diagram illustrates the selection and exclusion process of rheumatoid arthritis patients assessed for eligibility for infliximab therapy. All patients presented with active RA at baseline despite prior DMARD therapy, which supported escalation to biological treatment. The median duration of prior DMARD exposure was ≥6 months, with methotrexate as the standard background therapy. As shown in Figure 1, out of 68 patients initially screened, 16 were excluded based on predefined criteria (other autoimmune diseases, active infections, diabetes mellitus, severe hypertension, incomplete laboratory data, or corticosteroid therapy >7.5 mg/day). Baseline clinical assessment confirmed active RA in all patients before biological treatment initiation. All participants were seropositive for rheumatoid factor (RF) and/or anti-CCP antibodies. The remaining 52 patients were included in the study and received infliximab 3 mg/kg at weeks 0, 2, 6, and 8, in combination with methotrexate 15 mg once a week and folic acid 5 mg the next day. Monthly monitoring was performed throughout the study, and no patients were lost to follow-up. Laboratory analyses were conducted at baseline and after six months of therapy, including measurements of CBC, CRP, NLR, and SII. Statistical analysis was performed using Shapiro–Wilk, Kolmogorov–Smirnov, and Anderson–Darling tests to assess normality before comparing pre- and post-treatment data.

The patients were given infliximab at a dose of 3 mg/kg body mass at weeks 0, 2, 6, and 8. All patients took methotrexate and folic acid as part of their regular treatment.

The patients’ blood samples were collected twice to measure biochemical parameters: first before receiving the biologic therapy, known as the pre-treatment period, and again six months later, known as the post-treatment phase. A Sysmex XN-1000 analyzer (Sysmex Corporation, Hyogo 651-0073, Kobe, Japan) was used to evaluate the complete blood count (CBC) values. CRP was measured using immunoturbidimetry on the Roche Cobas.

The study’s parameters were derived using the following expressions: NLR = neutrophil count/lymphocyte count, PLR = platelet count/lymphocyte count, and SII = (neutrophils × platelets)/lymphocytes. The baseline and follow-up measurements for these indices were calculated.

The investigator analyzed paired measurements collected on the same subjects before (Pre) and after (Post) therapy for four inflammatory biomarkers (NLR, PLR, SII, and CRP). Data was imported from a single Excel worksheet, and calculations were performed in MATLAB (R2024b) using a reproducible script developed for this project.

Disease activity was assessed using the DAS28, calculated with CRP. Patients were classified into four standard disease activity categories: remission (DAS28 < 2.6), low disease activity (2.6–3.2), moderate disease activity (3.2–5.1), and high disease activity (>5.1). The number of patients in each category was determined separately for the pre-treatment and post-treatment groups. To evaluate whether therapy was associated with a change in the distribution of disease activity states, a chi-square test of independence was applied to compare the categorical DAS28 distributions between the two groups. This approach allows assessment of treatment-related shifts across clinically relevant disease activity categories without requiring assumptions of normality.

Prior to analysis, rows with missing values in either member of a Pre–Post pair were excluded listwise for that parameter, preserving the within-subject pairing. Descriptive summaries were derived for each biomarker, including mean, median, SD, MAD, and paired difference (D = Post − Pre). To assist distributional diagnostics, the investigator produced histograms of inflammatory biomarkers for pre and post circumstances with a fitted Gaussian density and normal QQ-plots comparing the empirical quantiles of D to those of a Gaussian with the same mean and SD. These visuals provide a quick visual confirmation of the Gaussian modeling assumption that underpins parametric paired inferences.

The validity of the parametric paired t-test is based on the approximate normalcy of the paired differences, not the normality of Pre or Post individually. Therefore, the investigator tested D using three complementary procedures: The Shapiro–Wilk (SW) test was applied to D and implemented in MATLAB using the community add-on function swtest.m [15]. SW is usually the most powerful omnibus test for small to intermediate samples, and it is particularly sensitive to deviations from normality in the middle of the distribution. The Kolmogorov–Smirnov (KS) test is performed against a normal reference with mean and standard deviation. KS compares the empirical distribution function of D to the cumulative distribution function of the reference Gaussian, providing a clear global goodness-of-fit diagnostic. The Anderson–Darling (AD) test for normality prioritizes the tails over KS and SW. This characteristic enhances SW’s center sensitivity and provides against tail-driven deviations that could affect t-based inference.

All tests were two-sided, with a nominal significance threshold of *α* = 0.05. Using three tests with varied sensitivities raises confidence that any subsequent parametric inference is based on a good approximation to Gaussianity. The intention is not that all three tests must always be significant or non-significant in perfect agreement, but that when combined with the histogram and QQ-plot, they support the normality assumption for D.

To test the null hypothesis against the two-sided alternative, a paired t-test was used for each biomarker, assuming adequate normality of D. The paired t-test is optimal under Gaussian D and it provides analysis of the two-sided *p*-value. The study also provides a 95% confidence interval for the mean pre-post difference as well as Cohen’s within-subject effect sizes. This study utilized a pre–post within-subject design, in which each participant served as their own control, enabling a direct comparison between pre- and post-treatment biomarker values. Therefore, the reductions observed in inflammatory markers should be interpreted as associations with infliximab exposure rather than definitive causal treatment effects. The sample size of this research allowed the identification of clear and consistent pre–post differences in inflammatory biomarkers. The statistical approach was tailored to this cohort size, including rigorous normality testing and paired comparisons to ensure objective and robust interpretation of the observed changes.

## 3. Results

**Figure 2:**
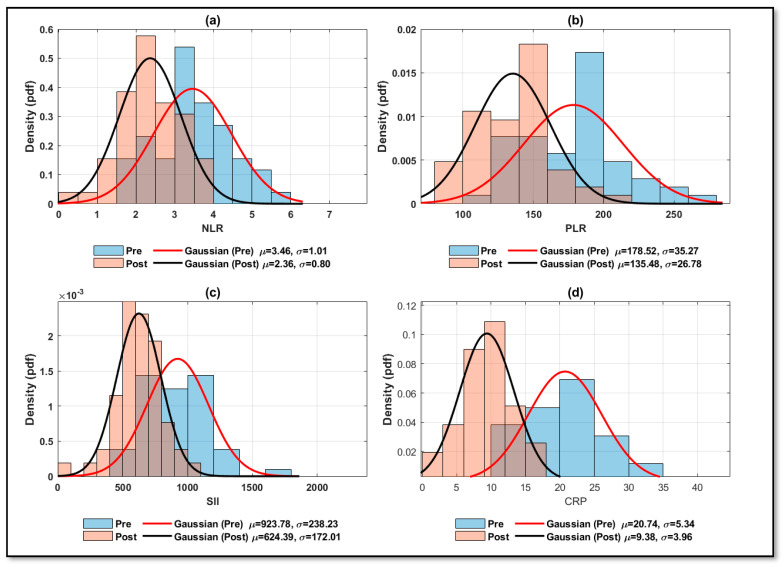
Histogram with fitted Gaussian for Pre and Post treatment: (**a**) NLR, (**b**) PLR, (**c**) SII, and (**d**) CRP.

To acquire a clearer and more visual perception of the result biomarkers under study, the data were prepared and presented in Figure 2. It presents pre- and post-treatment results for NLR, PLR, SII, and CRP as a density or probability density function (PDF) of histograms fitted with Gaussian curves. In general, all parameters under study exhibit comparable patterns; a leftward shift in the post-treatment distribution indicates lower values after therapy.

Across all four subplots of Figure 2, the post-treatment peak shifts left relative to the pre-treatment peak, indicating lower values after therapy and consistent with a treatment effect. The distribution after therapy is similarly more clearly defined, tighter, and less dispersed than the distribution before treatment. Among the four panels, CRP provides the strongest visual evidence of a post-treatment decrease.

The histogram and Gaussian data analysis show that the apparent value of improvement is about CRP > PLR > SII > NLR. All four biomarkers exhibit a leftward post-treatment shift, indicating reductions after therapy was taken, with CRP showing the greatest significant drop and NLR the least. The Gaussian overlays fit nicely into each panel, offering a clear and understandable visual overview of the pre-to-post shift.

**Figure 3:**
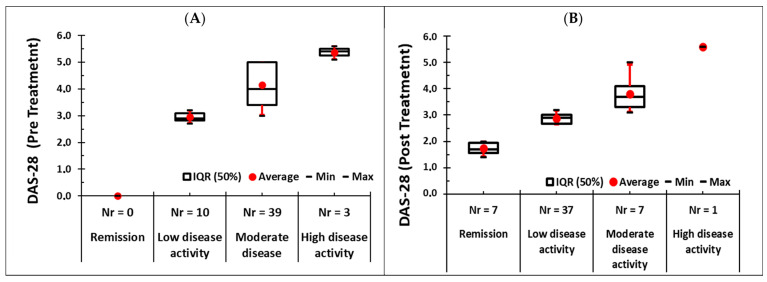
DAS28 changes (**A**) pre-treatment and (**B**) post-treatment.

Figure 3 illustrates the distribution of DAS28-CRP values across disease activity categories before and after treatment. In the pre-treatment panel (Figure 3A), patients were predominantly concentrated in the moderate and high disease activity groups, as reflected by higher median and mean DAS28 values. The moderate disease activity group showed a wide interquartile range and a broad 90% range, indicating substantial heterogeneity in inflammatory burden prior to therapy. The high disease activity group displayed consistently elevated DAS28 values, with both median and mean above 5, reflecting severe active disease. In contrast, no patients were observed in remission before treatment.

Following therapy (Figure 3B), a pronounced shift toward lower disease activity was observed. The remission and low disease activity groups showed low median DAS28 values with narrow interquartile and 90% ranges, indicating stable disease control in these categories. The moderate disease activity group demonstrated a clear reduction in both median and mean DAS28 compared with pre-treatment, accompanied by a compression of the distribution, reflecting a more homogeneous and lower inflammatory state. Only a single patient remained in the high disease activity category, represented by an isolated high mean value and wide min–max range.

Across all categories, the red circles (mean values) and horizontal median lines confirm a downward shift in DAS28 after therapy, while the reduced interquartile and 90% ranges indicate decreased variability in disease activity. The contraction of the distributions and the movement of patients toward remission and low disease activity provide strong graphical evidence of a substantial therapeutic effect.

In accordance with best practices for paired data, the paired differences pre- and post-results were analyzed using three complementary tests: Shapiro–Wilk (SW), Kolmogorov–Smirnov (KS), and Anderson–Darling (AD). A paired t-test was used to analyze differences that were close to normal. The paired *t*-tests provide two-sided *p*-values, 95% confidence intervals (CIs) for the mean difference, and Cohen’s, within-subject effect size. The displays for each parameter include a histogram of differences with a fitted normal curve and a QQ-plot to demonstrate the normality assumption.

For all four parameters, the analysis indicates that the difference scores post-Pre fulfill normalcy by all three tests SW, KS and AD, justifying the use of the paired t-test. The QQ-plots place most points near to the reference line, with only minor tail deviations typical for samples of this size.

**Figure 4:**
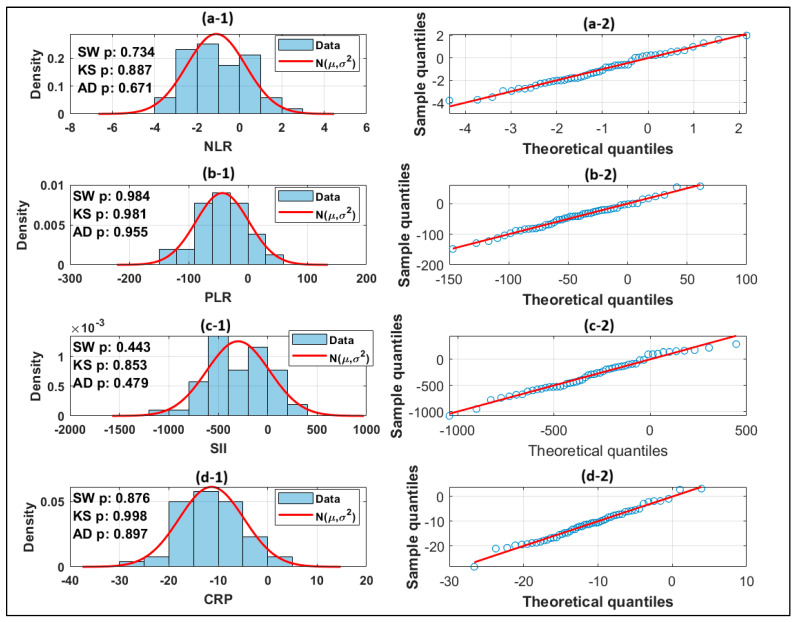
Statistical calculations, (**a-1**) QQ plot for NLR, (**a-2**) Theoretical quantile for NLR, (**b-1**) QQ plot for PLR, (**b-2**) Theoretical quantile for PLR, (**c-1**) QQ plot for SII, (**c-2**) Theoretical quantile for SII, (**d-1**) QQ plot for CRP, and (**d-2**) Theoretical quantile for CRP. The blue circles present experimental measurements, and the red line is theoretical calculation based on measurements.

The study found a significant reduction in NLR after treatment, with the mean for Pre = 3.46 and the mean for Post = 2.36, resulting in a mean difference of −1.096. With normality satisfied, the paired t-test shows a highly significant change: *p* = 6.48 × 10^−7^. The 95% confidence interval for −1.483 and −0.708 for low and high, respectively, excludes zero. The effect size based on Cohen’s calculation resulted in −0.788, which is typically interpreted as a large within-subject effect; these data are presented on Table 1. The direction is consistent with an anti-inflammatory post-treatment change. The NLR figure shows this pattern: the difference histogram closely matches the fitted normal curve, and the QQ-plot points are along the diagonal as presented in Figure 4(a-1,a-2).

Therapy provides a clear and statistically significant reduction in all three inflammatory indicators (PLR, SII, and CRP). PLR, SII, and CRP show an average decrease of −43.04, −299.39, and −11.36, respectively. All the difference values matched the normality assumptions for statistical methods SW, KS, and AD. Paired *t*-tests show significant changes (*p* < 10^−8^) for all parameters, with confidence intervals excluding zero and effect sizes ranging from large to extremely large. For more information, check Table 2.

The QQ plots of the differences D=Post − Pre for NLR, PLR, SII, and CRP in Figure 4 show that the points overlap closely with the 45° reference line across the middle quantiles, showing nearly normal distributions of D. Only minor tail departures are visible, with no persistent S-shape, no obvious fan-out, and no pattern indicating influential outliers. These diagnostics confirm the normalcy assumption for D in all four biomarkers, validating the use of the paired t-test and 95% confidence intervals presented in the results.

## 4. Discussion

The present findings demonstrate a marked therapeutic effect, as reflected by both DAS28-CRP distributions and categorical shifts in disease activity. Before treatment, the majority of patients were classified as having moderate or high disease activity (42 of 52 patients; 80.7%), with no patients in remission, indicating a substantial inflammatory burden. Following therapy, this pattern changed dramatically: 44 of 52 patients (84.64%) achieved either remission or low disease activity, while only 8 patients (15.4%) remained in moderate or high disease activity, as illustrated in Figure 3. This redistribution toward lower disease activity states is consistent with a strong reduction in systemic and joint-level inflammation. The change in DAS28 categories was statistically significant according to the Chi-square test (χ^2^ = 52.1, df = 3, *p* < 0.001), indicating that the improvement after treatment was not due to chance. Patients classified as having low disease activity at baseline according to DAS28 showed values ranging from 2.7 to 3.2, with a mean of 2.95 and a standard deviation of 0.17. This narrow distribution indicates that all ten patients were clustered close to the threshold for moderate disease activity. In addition, patients in this subgroup presented radiographic evidence of structural joint damage, elevated inflammatory markers, and relevant extra-articular manifestations, including gastrointestinal complaints. Based on this combined clinical and biological profile, the multidisciplinary clinical board justified the initiation of infliximab therapy. Furthermore, published evidence supports the use of biologic agents in patients classified as having low DAS28 when residual inflammatory activity, structural progression, or a high risk of disease worsening is present [16,17].

Paired differences in all four biomarkers (NLR, PLR, SII, and CRP) pass Shapiro–Wilk, Kolmogorov–Smirnov, and Anderson–Darling tests, indicating the validity of paired *t*-tests. Each parameter demonstrates a statistically significant reduction following therapy, with large or very large within-subject effect sizes, and all 95% confidence intervals for mean differences exclude zero. Overall, the evidence suggests that the medication had a clear and significant effect in decreasing systemic inflammatory markers in this population.

Statistically significant reductions in all inflammatory indicators were observed after six months of infliximab treatment. The NLR decreased from 3.7 to 2.4, PLR from 178 to 132, SII from 890 to 630, and CRP from 20.4 mg/L to 9.2 mg/L (all *p* < 0.001). It is worth noting that individual patient reaction varies, with approximately 15% of patients experiencing little or no drop in SII and CRP levels, indicating possible secondary non-responsiveness. Alternate biologics or combination therapy may be useful for these patients. The basis of a future analysis will be to examine whether the type of response varies in relation to the comorbidity of metabolic syndrome, anemia, or smoking history. Such patterns support why SII would be an evolving biomarker capable of detecting not only complete biologic inefficacy, but also partial biologic inefficacy. Also, it might be beneficial to draw the trajectories of changes in each patient and identify a possible pattern that can be used to build a model for machine learning.

Gender subgroup analysis revealed substantial reductions in all indices for both males and females. However, females had somewhat higher pre-treatment CRP levels (21.2 ± 6.4 mg/L) than males (19.1 ± 6.9 mg/L). The medication, however, caused a similar drop in both sexes.

This study evaluated changes in hematological inflammatory indices (NLR, PLR, SII) and their relationship with CRP in patients with rheumatoid arthritis (RA) undergoing infliximab therapy. The findings show that the indices significantly declined at six months of treatment, and this was accompanied by a sharp decline in the CRP levels. These findings support the utility of NLR, PLR, and SII as complementary tools to monitor systemic inflammation in RA. Another study from the UK reported that NLR and PLR were strongly associated as inflammatory markers in rheumatic disease monitoring [11]. Our data are congruent with these results and continue to contribute to the utility of these indices in the infliximab-treated population, a cohort that is otherwise not well-represented in inflammatory biomarker validation research.

The biological reasoning behind such indices should be brought up as well. Increased neutrophil reflects acute phase immune action, and oxidative stress acts as a promoting factor for joint destruction. On the other hand, lymphopenia, which is common in active RA, denotes inhibition of adaptive immune response. Increasing numbers of platelets, as reflected by PLR and SII, are driven by the effects of thrombopoietin triggered by interleukin-6 and other cytokines. Such mechanisms suggest that composite indices, such as SII, can measure wider dimensions of immune dysregulation than CRP alone.

However, there are limitations to the study. To begin with, the standardization of clinical indices such as DAS28 precludes comparison with clinical remission status. Second, the fairly short follow-up time does not allow for assessing the stability of biomarkers over a long period of time. Third, it is a single-center study carried out in Kosovo, and as a result, generalizability is limited. Finally, despite the use of CRP as the comparator biomarker, there still needs to be a substantial validation of the endpoints in terms of ESR and other imaging endpoints like power Doppler ultrasound.

Notwithstanding the limitations mentioned, the study provides important evidence to be utilized in favor of integrating NLR, PLR, and SII in RA observation systems. They are useful because of their cost-effectiveness, reproducibility, and availability in both resource-rich and resource-limited clinical practice. In addition to providing application in clinical practice, such hematologic indices can have predictive application in personalized medicine as well. Indicatively, those patients who are clinically in remission but still have a high SII might still have some inflammation in the body that is only visible on MRI or through ultrasound. These revelations could instruct clinicians to reduce therapy more prudently or watch out for subclinical activity. Equally, it is also possible that machine learning models can be used to predict the risk of flares by combining this predictive model with serial hematologic data.

The integration of these indices into electronic health records (EHRs) could offer real-time dashboard monitoring of disease activity, particularly in remote or telemedicine settings. This may be particularly revolutionary in low-resource settings where multiple imaging or testing of CRP is impossible. Lastly, the study of these indices in seronegative RA or with extra-articular involvement may reveal less obvious patterns of inflammation that would not be detected by established disease activity instruments.

Among the observed correlations, NLR, PLR, and SII with CRP suggest that this aspect shows the presence of an underlying inflammatory status. It is important to note that SII showed the most significant correlation with CRP, probably because there was the inclusion of neutrophil and platelet response in SII. Such hematological and biochemical alterations are consistent with the infliximab mechanism of action by inhibiting TNF-alpha and adjusting the activity of immune cells.

Our results can be aligned with several recent research projects. A study by Enginar et al. [18] in patients with active RA showed significant reductions in NLR following anti-TNF therapy, reinforcing the value of these indices as dynamic markers of treatment response. Similarly, Mainland [19] emphasized the clinical relevance of tracking composite inflammatory indices in RA management.

SII is comparatively new to the RA world, but it still has demonstrated potential in forecasting disease activity and outcomes. It combines neutrophil, thrombosis, and lymphopenia, which are all characteristics of chronic inflammation. Other studies have shown its predictive role in systemic lupus erythematosus, cardiovascular risk, and cancer prognosis [20,21,22].

The advantage of these indices is that they are based on routine CBC testing; therefore, they are cheap and easily available even where CRP testing is not feasible. Their incorporation into daily clinical work could potentially aid clinicians in monitoring inflammatory status in real time without any expensive biomarker profiles.

However, certain limitations must be recognized. To begin with, this research did not involve a control sample that did not receive infliximab. Finally, the sample was small, and only one center was included, which might prevent generalization.

## 5. Conclusions

Following six months of infliximab therapy in individuals with rheumatoid arthritis, this study found a significant decrease in all four inflammatory biomarkers: NLR, PLR, SII, and CRP. The paired differences had approximately normal distributions, as confirmed by the Shapiro–Wilk, Kolmogorov–Smirnov, and Anderson–Darling tests, allowing for robust parametric analysis. Statistical methods concluded a leftward shift in biomarker distributions after therapy, with CRP showing the greatest reduction, followed by PLR and SII, and a slight decrease in NLR. These data demonstrate a constant reduction in systemic inflammation and support the use of CBC-derived indices as cost-effective methods for evaluating treatment response.

Future studies should attempt to increase the sample size and include multicenter cohorts to improve generalizability. Longitudinal studies could evaluate biomarker stability over time, while subgroup analysis could reveal differences in responses based on comorbidities.

## Figures and Tables

**Table 1:** Demographic characteristics of patients under the study.

Characteristic	All Patients (N = 52)
Age, years—mean ± SD	52.3 ± 12.7
Female %	57.7
Male %	42.3
BMI, kg/m^2^—mean ± SD	23.4 ± 2.7
Disease duration, years—median (min-max)	4.1 (2.3–6.1)
Disease Activity Categories	
Remission	0
Low	10
Moderate	39
High	3
Marital Status	
Single %	18.9
Married %	72.7
Divorced %	8.4
Education	
Primary school %	28.7
Secondary School %	57.7
University %	13.6
Occupation	
Unemployed %	35.9
Employed %	52.6
Retired %	11.5

**Table 2:** Statistical parameters for inflammatory biomarkers.

Biomarkers	*p*_Value	Test Stat	CI 95_lo	CI 95_hi	Cohens Effect Size
NLR	6.48 × 10^−7^	−5.680	−1.483	−0.708	−0.788
PLR	5.77 × 10^−9^	−6.984	−55.411	−30.667	−0.969
SII	1.20 × 10^−8^	−6.783	−388.007	−210.782	−0.941
CRP	0.00 × 10^+0^	−12.545	−13.177	−9.541	−1.740

## Data Availability

Microsoft Excel and MATLAB (R2024b) were used to generate figures and/or statistical analyses. The original contributions presented in this study are included in the article. Further inquiries can be directed to the corresponding author.

## Abbreviations

The following abbreviations are used in this manuscript:

NLR	Neutrophil-to-Lymphocyte Ratio
PLR	Platelet-to-Lymphocyte Ratio
SII	Systemic Immune Inflammation Index
CRP	C-reactive Protein
ESR	Erythrocyte Sedimentation Rate
RA	Rheumatoid Arthritis
CBC	Complete Blood Count
DAS28	Disease Activity Score in 28 joints
SW	Shapiro–Wilk
KS	Kolmogorov–Smirnov
AD	Anderson–Darling
WHO	World Health Organization
DALYs	Disability-Adjusted Life Years
ACR	American College of Rheumatology
EULAR	European Alliance of Associations for Rheumatology
DMARD	Disease-Modifying Anti-Rheumatic Drug
SD	Standard Deviation
QQ	Quantile–Quantile
MAD	Mean Absolute Deviation
